# Subclinical hypothyroidism and anxiety may contribute to metabolic syndrome in Sichuan of China: a hospital-based population study

**DOI:** 10.1038/s41598-020-58973-w

**Published:** 2020-02-10

**Authors:** Rui-cen Li, Lingyun Zhang, Han Luo, Yali Lei, Li Zeng, Jingqiang Zhu, Huairong Tang

**Affiliations:** 1 Health Promotion Center of West China hospital, Sichuan University, Chengdu, P.R. China; 2 Thyroid & parathyroid Surgery of West China hospital, Sichuan University, Chengdu, P.R. China

**Keywords:** Metabolic syndrome, Population screening, Risk factors

## Abstract

The prevalence of Metabolic syndrome (MetS) in Sichuan of China has not yet been estimated. Meanwhile the association among anxiety, subclinical hypothyroidism (SCH) and MetS was less well-studied. The data was retrieved retrospectively from Health Promotion Center of West China Hospital database between 2014 and 2017. Internal validation by randomizing into training and testing panel by 9:1 and external validation with National Health and Nutrition Examination Survey (NHNES) were conducted. 19006 subjects were included into analysis, and 3530 (18.6%) of them were diagnosed with MetS. In training panel, age, sex (male), SCH (presence), SAS score, alcohol (Sometimes & Usual) and smoking (Active) were identified as independent risk factors for MetS, which was confirmed in testing panel internally. NHNES data validated externally the association between free thyroxine (fT4) and MetS components. The C-indices of predicting MetS nomogram were 0.705 (95% CI: 0.696–0.714) and 0.728 (95% CI: 0.701–0.754) in training and testing panel respectively. In conclusion, MetS prevalence was 18.6% in Sichuan. SCH and anxiety may be associated with MetS independently. A risk scale-based nomogram with accurate and objective prediction ability was provided for check-up practice, but more cohort validation was needed.

## Introduction

Metabolic syndrome (MetS) is characterized by a cluster of type 2 diabetes, cardiovascular disease risk factors, including hyperglycemia, central obesity, dyslipidemia and hypertension. It was firstly proposed in 1998 and finalized in 1999 by the WHO consultation group^1^. Prevalence of MetS roars worldwide in recent decades and becomes a major public health concern. Over 20% of adults are beset by it in the United States according to the result of National Health and Nutrition Examination Survey (NHNES)^2^. In a meta-analysis of 35 published papers, the pooled prevalence of MetS in mainland China is 24.5% (range: 13.15%, 46.3%)^3^. However, no specific data of Sichuan province were reported, which locates in the southwest of China with nearly 100 million population.

Meanwhile, MetS-related risk factors vary in previous different researches, yet consensus was reached that variables-age, body mass index, socioeconomic (household income, education level *et al*.) and lifestyle characteristics (physical activity, smoking *et al*.) were defined as risk factors^4^. Recently, a systematic review and meta-analysis revealed anxiety is associated with MetS^5^. Yang L’s *et al*. study indicated subclinical hypothyroidism (SCH) has a significant relation with higher risk of MetS^6^. Notably, thyroid hormone often relates to anxiety. Yoshifumi Koshino *et al*. found the association between clinical features of anxiety and thyroid hormone level^7^. And it may be explained by the physiological interaction function between nervous and endocrine system partly^8,9^. These evidences implied the potential of anxiety and SCH as independent risk factors for MetS, which has not yet been investigated and discussed in an integrated way. Besides, subclinical hypothyroidism affects up to 10% of the population, with the highest prevalence among women and elderly individuals^10,11^. It gradually becomes a public concern in population. Therefore, we orchestrated to explore the MetS prevalence in SC province in the hospital-based population study, and investigated the relationship among anxiety, SCH and MetS.

## Methods

### Study design and subjects

This hospital-based population study was conducted in Health Promotion Center of West China Hospital between 2014 and 2017. West China hospital is a regional tertiary medical center in Sichuan (SC) province with two affiliated branches in two cities of SC. The health promotion center provides physical examination for more than 60,000 subjects every year.

All subjects with complete clinical (weight, height, blood pressure *et al*.), biochemical (thyroid hormone, fasting glucose, triglyceride *et al*.), computer-based psychological and sleep quality survey (Self-Rating Anxiety Scale-SAS, Self-Rating Depression Scale-SDS and Pittsburgh Sleep Quality Index-PSQI) between 2014 and 2017 were included. In the present study, subjects with incomplete information and overt hypo/hyperthyroidism were excluded from the final analysis.

The study protocol was approved by the medical ethical review committee of the West China hospital. All study participants provided written informed consent to indicate their agreement for the clinical data to be used in clinical research and publication. The methods were carried out in accordance with the Declaration of Helsinki and the guidelines of the Ethical Committee of the West China Hospital (Chengdu, China).

All included subjects were randomly divided into 2 panels (training and testing panel) by 9:1 ratio using the sealed envelope method (https://www.sealedenvelope.com/). The training and testing panel included 17108 and 1898 subjects respectively. Furthermore, to confirm the bona fide result of the present research, National Health and Nutrition Examination Survey (NHNES) data was applied to validate externally.

### Physical examination and lipid profile

Height, weight, blood pressure and waist circumference with subjects wearing light clothes were measured by trained staff of Health Promotion Center in a private room. Body mass index (BMI) was calculated as weight in kilograms divided by the square of height in meters. An automated clinical chemistry analyzer Hitachi 7180 (Hitachi Ltd, Tokyo, Japan) was used to measure lipid profile (fasting glucose, HDL and triglyceride *et al*.).

### Stratification of cigarette and alcohol consumption

Each subject was inquired whether they smoke (Never or Quit or Current). For subjects who reported Quit, how long they have quit was asked to further specify. In terms of alcohol consumption, it was subdivided into 4 category-Never/Quit/Sometimes/Usual. Sometimes defines the subject drinks less than three times per week here, and Usual means more than that.

### Brief assessment of psychological and sleep

In Health Promotion Center of West China hospital, psychological and sleep quality survey were conducted by computer-based method. Anxiety, depression and sleep quality were assessed by SAS, SDS and PSQI questionnaire respectively. Both SAS and SDS are 20-item self-report assessment devices built to measure anxiety and depression levels. With normalized score, subjects were grouped into 4 levels (Normal, Mild, Moderate and Severe anxiety/depression levels)^12,13^. The Pittsburgh Sleep Quality Index (PSQI), consisting of 19 individual items, is a self-report questionnaire that assesses sleep quality over a 1-month time interval, by which subjects were also classified into 4 levels (Excellent, Satisfied, Less Satisfied, Unsatisfied)^14^.

### MetS and SCH definitions

The diagnosis criteria of MetS in modified NCEP ATP III is the presence of any three of the following five risk factors: ➀ abdominal obesity (waist circumference >90 cm in men and 80 cm in women); ➁ a high triglyceride level (≥150 mg/dL [≥1.69 mmol/L]); ➂ a low HDL cholesterol level (<40 mg/dL [<1.03 mmol/L] for men and <50 mg/dL [<1.29 mmol/L for women]; ➃ high blood pressure (systolic ≥130 mm Hg or diastolic ≥85 mm Hg); ➄ a high fasting plasma glucose concentration (≥110 mg/dL). Individuals currently using specific anti-hypertension medications, oral hypoglycemic diabetes drugs or insulin met the criteria of high blood pressure or high fasting glucose. However, subjects who have previous hypertension or diabetes diagnosis but cannot report medications they are using specifically were not allocated to the high blood pressure or glucose group. Accordingly, SCH is biochemically defined as an elevated serum thyrotropin level in combination with a serum free T4 (thyroxine) level that is within the population reference range^15^.

### Statistical analysis

SPSS version 21 (SPSS Inc, Chicago, IL) was used in the data analysis. Variables were presented as the mean ± standard deviation or median (interquartile range) and compared by t-test or U-test. Chi-square test was performed to compare frequency for categorical variables. Based on the variables that were statistically significant in univariate analysis, multivariate analysis with logistic regression was conducted to identify independent risk factors for MetS. The results of the multivariate analysis were expressed as odds ratio and 95% confidence interval (CI). A Pearson correlation graph by adjusted dot plot was conducted to visualize the correlation between components of MetS and independent risk factors. R programming studio was used to randomize group and graph. P value less than 0.05 indicates significance.

## Results

**Figure 1:**
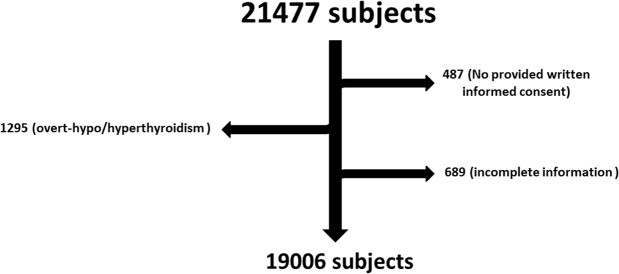
Subjects inclusion and exclusion flowchart. In all, 21477 subjects underwent complete physical examination in Health Promotion Center of West China Hospital from 2014 to 2017. 1295 of them were diagnosed with overt hypo/hyperthyroidism and were removed from final analysis. 487 and 689 were also excluded because of lacking written informed consents and incomplete information, respectively.

**Figure 2:**
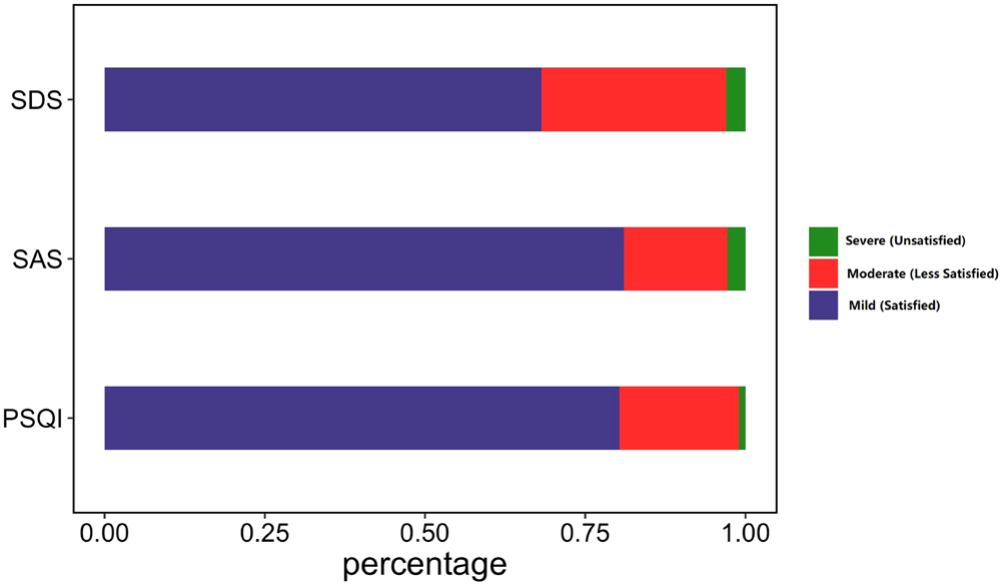
Psychological condition and sleep quality distribution diagram. The blue, red and green bars were used to represent three different levels for SDS, SAS and PSQI scores, respectively. The length of bars stood for the percentage of relevant levels in all aberrant groups, in which Normal/Excellent level was excluded for diagram beautifying. For SDS, Mild, Moderate and Severe groups accounted for 68.21%, 28.75% and 3.04%; for SAS, the proportions were 81.06%, 16.13% and 2.81%; and for PSQI, 80.33%, 18.62% and 1.05% subjects reported their sleep quality as Satisfied, Less Satisfied and Unsatisfied, respectively.

In all, 21477 subjects underwent complete physical examination in Health Promotion Center of West China Hospital from 2014 to 2017. 1295 of them were diagnosed with overt hypo/hyperthyroidism and were removed from final analysis. 487 and 689 were also excluded because of no provided written informed consent and incomplete information, respectively. Consequently, a total of 19006 subjects with a mean age of 42.92 ± 9.08 (18–80) were finally included into the analysis. A subjects inclusion and exclusion flowchart for the study was shown in Fig. 1. Gender ratio of man and woman was 56.8% vs 43.2%, and mean of free thyroxine and thyroid stimulating hormone (TSH) was 16.67 ± 2.52 pmol/L and 2.94 ± 2.74 mU/L respectively. 18.6% (3530/19006) and 14.8% (2811/19006) of subjects had MetS and SCH respectively. The distribution of psychological condition and sleep quality was shown in Fig. 2.

### Data characteristics of training panel and testing panel

In order to validate the result of risk factor identification, 19006 subjects were randomized into two panels with 9:1 ratio-training panel (n = 17108) and testing panel (n = 1898). Mean age, gender ratio, thyroid hormone and blood pressure *et al*. were similar between both panels, and no significant difference was found between two panels in any category. The Prevalence of SCH in two panels was 14.5% and 14.6% respectively, and the prevalence of MetS was 18.5% and 19.0% respectively **(**Table 1**)**. Therefore, the baseline characteristic of two panels was comparable.

**Table 1:** Data characteristics in training panel and testing panel.

Characteristics	Training panel (n = 17108)	Testing panel (n = 1898)	p value
**Age**			0.385
≥45	7771 (45.42)	882 (46.47)	
<45	9337 (54.58)	1016 (53.53)	
**Sex**			0.708
Female	7405 (43.3)	813 (42.8)	
Male	9703 (56.7)	1085 (57.2)	
**SCH**			0.853
Presence	2533 (14.8)	278 (14.6)	
Absence	14575 (85.2)	1620 (85.4)	
**MetS**			0.598
Presence	3169 (18.5)	361 (19.0)	
Absence	13939 (81.5)	1537 (81.0)	
**Depression score stratification**			0.898
None - Mild	16093 (94.1)	1784 (94.0)	
Moderate - Severe	1015 (5.9)	114 (6.0)	
**Anxiety score stratification**			0.064
None - Mild	16624 (97.2)	1830 (96.4)	
Moderate - Severe	484 (2.8)	68 (3.6)	
**Sleep quality stratification**			0.605
Excellent - Satisfied	15688 (91.7)	1747 (92.0)	
Less satisfied - Unsatisfied	1420 (8.3)	151 (8.0)	
**Alcohol**			0.814
Never & Quite	8729 (51.0)	963 (50.7)	
Sometimes & Usual	8379 (49.0)	935 (49.3)	
**Smoking**			0.94
Never & Quite	12452 (72.8)	1383 (72.9)	
Current	4656 (27.2)	515 (27.1)	
**Age (years)**	42.90 ± 9.07	43.07 ± 9.23	0.4393
**TSH (mU/L)**	2.94 ± 2.73	2.92 ± 2.80	0.7626
**fT4 (pmol/L)**	16.67 ± 2.54	16.71 ± 2.34	0.5119
**Triglyceride (mmol/L)**	1.60 ± 1.32	1.59 ± 1.27	0.7533
**HDL (mmol/L)**	1.4060 ± 0.39	1.40 ± 0.40	0.5259
**FG (mmol/L)**	5.14 ± 1.14	5.17 ± 1.17	0.2780
**SBP (mmHg)**	116.00 ± 15.28	116.24 ± 15.15	0.5159
**DBP (mmHg)**	73.54 ± 10.85	73.65 ± 10.91	0.6754
**BMI (Kg/m**^**2**^**)**	23.76 ± 3.31	23.87 ± 3.42	0.1710
**SDS score**	43.17 ± 10.74	43.13 ± 10.82	0.8777
**SAS score**	40.71 ± 8.57	40.68 ± 8.71	0.8851

Values are expressed as N (%) or mean ± SD. TSH - thyrotropin, thyroid stimulating hormone; fT4 - free thyroxine; HDL - high density lipoprotein; FG - free glycerol; SBP - systolic blood pressure; DBP - diastolic blood pressure; BMI - body mass index; SCH - subclinical hypothyroidism.

### Risk factors for MetS

In univariate analysis, age, sex, presence of SCH, alcohol consumption (Sometimes & Usual) and smoking (Active) were identified as risk factors for Mets in training panel. Likewise, the same risk factors were identified in testing panel **(**Table 2**)**.

**Table 2:** Univariate analysis for risk factors for MetS in training panel and testing panel.

	Training panel		Test panel	
	OR (95% CI)	p value	OR (95% CI)	p value
Age	1.054 (1.048, 1.061)	<0.001	1.047 (1.029, 1.065)	<0.001
**Sex**				
male	2.341 (2.014, 2.721)	<0.001	2.010 (1.266, 3.190)	0.003
female	1 (reference)		1 (reference)	
**SCH**				
Presence	1.217 (1.056, 1.402)	0.007	1.556 (1.030, 2.351)	0.036
Absence	1 (reference)		1 (reference)	
SDS score	0.997 (0.990, 1.015)	0.482	0.997 (0.975, 1.019)	0.791
**Depression score stratification**				
None - Mild	1 (reference)		1 (reference)	
Moderate - Severe	1.000 (0.767, 1.304)	>0.999	0.762 (0.331, 1.754)	0.523
SAS score	1.005 (0.996, 1.015)	0.264	1.008 (0.981, 1.037)	0.561
**Anxiety score stratification**				
None - Mild	1 (reference)		1 (reference)	
Moderate - Severe	1.077 (0.744, 1.560)	0.693	1.547 (0.599, 3.998)	0.367
**Sleep quality stratification**				
Excellent - Satisfied	1 (reference)		1 (reference)	
Less satisfied - Unsatisfied	0.871 (0.710, 1.069)	0.187	0.746 (0.391, 1.426)	0.376
**Alcohol**				
Never & Quite	1 (reference)		1 (reference)	
Sometimes & Usual	1.147 (1.010, 1.303)	0.035	1.490 (1.011, 2.196)	0.044
**Smoking**				
Never & Quite	1 (reference)		1 (reference)	
Active	1.336 (1.191, 1.499)	<0.001	1.673 (1.190, 2.352)	0.003

By multivariate analysis, age [1.054 (1.048, 1.061), p < 0.001], sex (male) [2.337 (2.012, 2.714), p < 0.001], SCH (presence) [1.216 (1.056, 1.401), p = 0.007], SAS score [1.008 (1.001, 1.015), p = 0.028], alcohol (Sometimes & Usual) [1.147 (1.011, 1.303), p = 0.034] and smoking (Active) [1.333 (1.189, 1.494), p < 0.001] were identified as independent risk factors for MetS in training panel. Moreover, the result was confirmed in testing panel completely **(**Table 3**)**. Furthermore, multivariate analysis with logistic regression was conducted when using another ratio of 5:5-training panel (n = 9503) and testing panel (n = 9503), and similar result was observed **(**Supplementary Table 1 online**)**.

**Table 3:** Multivariate analysis for risk factors for MetS in training panel and testing panel.

	Training panel		Testing panel	
	OR (95% CI)	p value	OR (95% CI)	p value
Age	1.054 (1.048, 1.061)	<0.001	1.047 (1.029, 1.065)	<0.001
Sex (male)	2.337 (2.012, 2.714)	<0.001	2.023 (1.281, 3.195)	0.003
SCH (Presence)	1.216 (1.056, 1.401)	0.007	1.527 (1.042, 2.372)	0.031
SAS score	1.008 (1.001, 1.015)	0.028	1.012 (1.004, 1.031)	0.041
Alcohol (Sometimes& Usual)	1.147 (1.011, 1.303)	0.034	1.489 (1.011, 2.192)	0.044
Smoking (Active)	1.333 (1.189, 1.494)	<0.001	1.663 (1.186, 2.332)	0.003

### Correlation between components of MetS and independent risk factors

**Figure 3:**
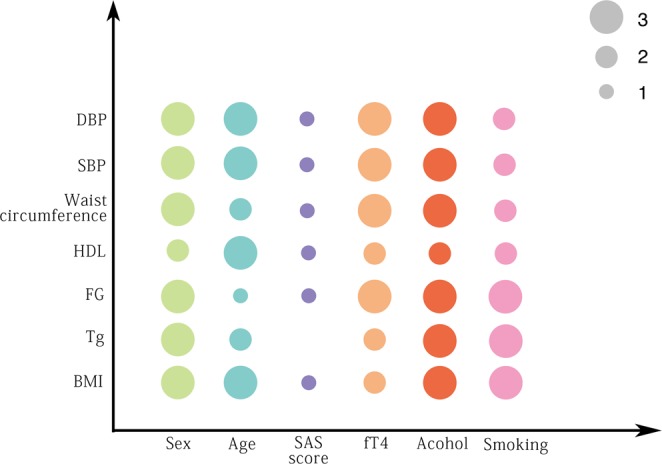
Pearson correlation graph between components of MetS and independent risk factors with adjusted dot plot. All independent risk factors and components of MetS were represented in transverse axis and vertical axis respectively. The presence of a color dot in the cross of vertical axes indicated an identified significant correlation between the two elements, and different size of the dot relates to corresponding range of Pearson’s correlation coefficient (r). Size 1, 2, 3 meant a r value range of 3 × 10^−4^ − 3 × 10^−3^, 3 × 10^−3^–3 × 10^−2^ and 3 × 10^−2^–3 × 10^−1^ respectively. Except SAS score did not correlate with triglyceride, all other components had reciprocal correlation significantly.

To visualize the correlation between components of MetS and independent risk factors, we graphed Pearson correlation by adjusted dot plot. As shown in Fig. 3, except SAS score did not correlate with triglyceride, all other components had reciprocal correlation significantly. In order to further confirm the correlation result, NHNES database was used as external validation. 2001–2002 NHNES analysis showed similar result. fT4 (free thyroxine) correlated with SBP, waist circumference, HDL, FG, TG and BMI significantly, p < 0.05. Gender, age, alcohol and smoking consumption correlated with nearly all components of MetS. Unfortunately, anxiety condition had no significant correlation with any component of MetS in NHNES database. It may be caused by adoption of different anxiety screening questionnaire tool.

### Prediction of the possibility of MetS

**Figure 4:**
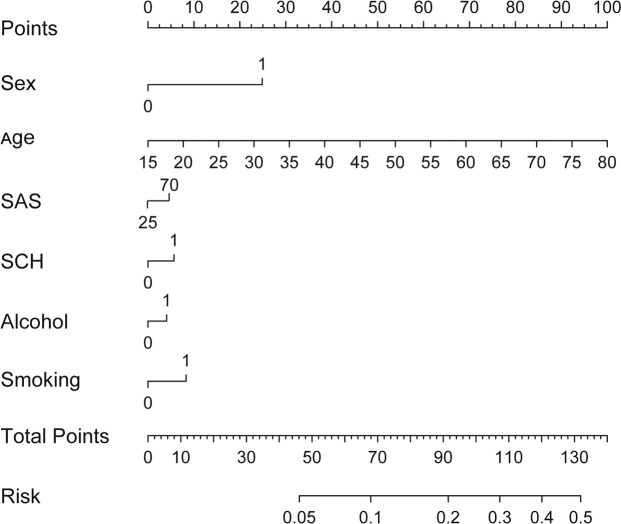
Nomogram for prediction of related risk factors and the possibility of MetS. All independent risk factors with statistical significance (sex, age, SAS score, SCH, alcohol and cigarette consumption) were included and unified into one risk factor MRRF (MetS-related risk factor). The 1st line is the standard scale compared to each downward line (2–7), by which a specific score of each line could be calculated. Then sum of 6 lines (scores) will be put on the 8^th^ line as the total points, and projected to the last line, which stands for the possibility of MetS. The C-indices of predicting MetS nomogram were 0.705 (95% CI: 0.696, 0.714) and 0.728 (95% CI: 0.701, 0.754) in training and testing panel respectively, indicating that the proposed nomogram of MetS prediction performed well in all MetS subjects.

All independent risk factors with statistical significance were included in the nomogram. Thus, sex, age, SAS score, SCH, alcohol and cigarette consumption were unified into one risk factor MRRF (MetS-related risk factor). As shown in Fig. 4, the 1st line is the standard scale compared to each downward line (2–7), by which a specific score of each line could be calculated. Then sum of 6 lines (scores) will be put on the 8^th^ line as the total points, and projected to the last line, which stands for the possibility of MetS. The C-indices of predicting MetS nomogram were 0.705 (95% CI: 0.696, 0.714) and 0.728 (95% CI: 0.701, 0.754) in training and testing panel respectively, indicating that the proposed nomogram of MetS prediction performed well in all MetS subjects.

## Discussion

According to the data from the US National Health and Nutrition Examination Survey, more than 1 in 3 adults have MetS, and alarmingly, about 40% of adults aged 40 and older have MetS, which has been reported as a major contributor to the epidemic of cardiovascular and a serious public health concern^16,17^. SCH, regarded as the manifestation of early and mild thyroid failure, occurs in 4–20% of the adult population worldwide, and the risk of progression of subclinical hypothyroidism to overt hypothyroidism is approximately 2 to 6% per year^15^. Anxiety also causes a series of assignable socioeconomic and public health problems, thus a better-established identification and management system is urgently need^18^.

To our limited knowledge, it is the first research about MetS investigation with such a considerable sample size (19006 subjects) in single institution of China. West China hospital is the 2^nd^ ranked regional tertiary hospital located in Sichuan (SC) province offering medical check-up service for 60,000 subjects each year. Until the present study, no cross-section study has been conducted in SC, in which nearly 100 million population live. Generally, 18.6% of subjects had MetS, which is consistent with the pooled estimate of MetS prevalence in China-24.5% (13.6–46.3%)^3^.

MetS has been reported to have association with anxiety and thyroid hormone^5,6^. However, all the related researches focus on either psychological condition or thyroid hormone independently. The relation between anxiety and thyroid dysfunction was detected in a variety of previous studies, which may be attributed to the sensitivity of the central system function to thyroid hormone in part^19,20^. However, the reciprocal relation among thyroid hormone, psychological condition and MetS remains unexplainable. With complete information, the present study is the first one to identify SCH and anxiety are independent risk factors for MetS, which pointing out that mental screening may be beneficial in check-up, especially to subjects with MetS.

In the present study, it was proved that fT4 was associated with components of MetS, which was validated in NHNES database and consistent with previous studies^21,22^. Therefore, the thyroid function screening should be also paid attention in MetS subjects. In terms of the physiological function, thyroid hormones have a range of effects on energy homeostasis, lipid and glucose metabolism, and blood pressure, most of which are considered as important components of MetS, so it is reasonable that metabolic alterations could be observed in subjects with SCH^23–25^. On the other hand, Chang, C. H. *et al*. also found that Individuals with MetS are at a greater risk for developing SCH, which further verified the association between SCH and MetS, but more exact mechanism needed to be explored^26^.

However, anxiety score did not show any significant correlation with MetS components in NHNES database, though SAS score related to all components of MetS except triglycerides in West China hospital’s (WCH) database. The discrepancy may be mainly attributed to different screening instruments of anxiety in NHNES and WCH database (Generalized anxiety disorder module and Self-Rating Anxiety Scale respectively). As concerning the previous studies about the relation between anxiety and MetS, contradictory findings were observed and it was hard to draw a consistent conclusion^5^. Further prospective study is needed.

Besides of anxiety, depression is recognized as another kind of mood change or neuropsychiatric disorder. Chueire, V. B.’s *et al*. and Delitala, A. P.’s *et al*. studies confirmed thyroid hormone relates to depression, while some other articles concluded that no significant difference in depressive symptoms has been found between euthyroid and SCH patients^27–31^. Recently, Kim, M. D. *et al*. found that depressed individuals with SCH are more likely to meet the criteria for MetS, indicating the existence of the complicated relation among depression, SCH and MetS^32^. In the current study, we failed to identify depression as independent risk factor for MetS, which was in line with findings of some previous studies but in conflict with others^33–35^. To a great extent, this contradiction could be attributed to the differences in socioeconomics, cultural background and diagnostic criteria among various researches. More prospective and well-designed study is needed.

With respect to gender difference, in our study, multivariate analysis identified sex (male) [2.337 (2.012, 2.714), p < 0.001]) as one of independent risk factors for MetS. This result may be conflicted with some previous studies^36–38^. However, it was consistent with several European studies which had indicated lower prevalence in women than in men^39–42^. Meanwhile, Li R’s *et al*. meta-analysis indicated female was not a significant risk factor for MetS in mainland China^6^. The contradiction may be explained by the feature of hospital-based study, which could not completely reflect the nature nationwide. In all, the distinction of prevalence of MetS between sex varies in different geographic regions and remains controversial.

Our study also showed a combination of sex, age, SAS score, SCH, alcohol and cigarette consumption had a promising prediction ability of MetS, which was validated in both training and testing panels (with the C-indices of 0.705 and 0.728 respectively). Nomogram-based prediction model is a user-friendly screening tool for check-up practice.

Unfortunately, our study was limited by its retrospective and cross-section nature. More cohort or prospective study is needed to confirm the result of the present study. Though the risk factors have been validated in NHNES database externally, the prediction model needs further external validation.

## Conclusions

It was the first report about MetS prevalence (18.6%), in SC province in China. Thyroid dysfunction-subclinical hypothyroidism and anxiety were independently associated risk factors for MetS, indicating the importance of screening thyroid hormone and psychological condition in MetS subjects. Nomogram-based risk scale has an accurate and objective prediction ability.

## Supplementary information

Supplementary Information.

## Data Availability

The datasets generated during and/or analysed during the current study are available from the corresponding author on reasonable request.
