# Supplementary material for: Subclinical hypothyroidism and anxiety may contribute to metabolic syndrome in Sichuan of China: a hospital-based population study
Source: Sci Rep. 2020 Feb 10;10:2261. doi: 10.1038/s41598-020-58973-w (PMC7010766; doi:10.1038/s41598-020-58973-w)
Supplement: Supplementary file 1 — Supplementary Information. [file 41598_2020_58973_MOESM1_ESM.docx]

Supplementary information

**Subclinical hypothyroidism and anxiety may contribute to metabolic syndrome in** **Sichuan of** **China: a hospital-based population study**

**Rui-cen Li M.S^1^#; Lingyun Zhang M.D^2^#; Han Luo M.D/Ph.D^2^; Yali Lei B.S^1^; Li Zeng B.S^1^; Jingqiang Zhu M.D ^2^*, Huairong Tang B.S ^1^***

**1.** **Health Promotion Center of West China hospital, Sichuan University, P.R. China;**

**2. Thyroid & parathyroid Surgery of West China hospital, Sichuan University, P.R. China;**

**#authors contribute equally**

*** To whom correspondence should be addressed (Huairong Tang and Jingqiang Zhu). E-mail:** [**HuairongTang@163.com**](mailto:HuairongTang@163.com) **and** [**zjq-wkys@163.com**](mailto:zjq-wkys@163.com)

**Table 1 Multivariate analysis for risk factors for MetS in training panel and testing panel by 5:5**

|  | **Training panel** | | **Testing panel** | |
| --- | --- | --- | --- | --- |
|  | **OR (95% CI)** | **p value** | **OR (95% CI)** | **p value** |
| **Age** | 1.055 (1.048, 1.062) | <0.001 | 1.050 (1.044, 1.057) | <0.001 |
| **Sex (male)** | 2.665 (2.268, 3.131) | <0.001 | 2.683 (2.290, 3.142) | <0.001 |
| **SCH (Presence)** | 1.196 (1.025, 1.396) | 0.023 | 1.343(1.154, 1.563) | <0.001 |
| **SAS score** | 1.011 (1.004, 1.019) | 0.004 | 1.012 (1.005, 1.020) | 0.002 |
| **Alcohol (Sometimes& Usual)** | 1.215 (1.059, 1.394) | 0.005 | 1.199 (1.047, 1.372) | 0.009 |
| **Smoking (Active)** | 1.475 (1.303, 1.671) | <0.001 | 1.429(1.264, 1.616) | <0.001 |
